# Alteration of the Redox State with Reactive Oxygen Species for 5-Fluorouracil-Induced Oral Mucositis in Hamsters

**DOI:** 10.1371/journal.pone.0082834

**Published:** 2013-12-20

**Authors:** Fumihiko Yoshino, Ayaka Yoshida, Atsushi Nakajima, Satoko Wada-Takahashi, Shun-suke Takahashi, Masaichi Chang-il Lee

**Affiliations:** 1 Photomedical Dentistry Division, Department of Oral Science, Kanagawa Dental University Graduate School, Yokosuka, Kanagawa, Japan; 2 Division of Pharmacology, Department of Clinical Care Medicine, Kanagawa Dental University, Yokosuka, Kanagawa, Japan; 3 Dentistry of Circulation Control Division, Department of Oral Science, Kanagawa Dental University Graduate School, Yokosuka, Kanagawa, Japan; 4 Yokosuka-Shonan Disaster Health Emergency Research Center & ESR Laboratories, Kanagawa Dental University Graduate School, Yokosuka, Kanagawa, Japan; Istituto di Ricerche Farmacologiche Mario Negri, Italy

## Abstract

Oral mucositis is often induced in patients receiving cancer chemotherapy treatment. It has been reported that oral mucositis can reduce quality of life, as well as increasing the incidence of mortality. The participation of reactive oxygen species (ROS) in the pathogenesis of oral mucositis is well known, but no report has actually demonstrated the presence of ROS. Thus, the purpose of this study was thus to demonstrate the involvement of ROS and the alteration of the redox state in oral mucositis using an *in vivo* L-band electron spin resonance (ESR) technique. An oral mucositis animal model induced by treatment of 5-fluorouracil with 10% acetic acid in hamster cheek pouch was used. Lipid peroxidation was measured as the level of malondialdehyde determined by the thiobarbituric acid reaction. The rate constants of the signal decay of nitroxyl compounds using *in vivo* L-band ESR were calculated from the signal decay curves. Firstly, we established the oral mucositis animal model induced by treatment of 5-fluorouracil with acetic acid in hamster cheek pouch. An increased level of lipid peroxidation in oral mucositis was found by measuring malondialdehyde using isolated hamster cheek pouch ulcer. In addition, as a result of *in vivo* L-band ESR measurements using our model animals, the decay rate constants of carbamoyl-PROXYL, which is a reagent for detecting the redox balance in tissue, were decreased. These results suggest that a redox imbalance might occur by excessive generation of ROS at an early stage of oral mucositis and the consumption of large quantities of antioxidants including glutathione in the locality of oral mucositis. These findings support the presence of ROS involved in the pathogenesis of oral mucositis with anti-cancer therapy, and is useful for the development of novel therapies drugs for oral mucositis.

## Introduction

Currently, 5-fluorouracil (5-FU) is the most effective agent in the treatment of gastrointestinal cancer [1]. However, oral mucositis is frequently associated with the use of radiation and the administration of anti-cancer drugs, such as 5-FU, for the treatment of head and neck cancer [2]. In general, this oral mucositis can be painful, limiting oral intake, and acting as portals of entry for indigenous oral microbial flora [3]. In another study, difficulty eating and drinking was reported in nearly 90% of patients and resultant weight loss in approximately 85%. One-third also had difficulty with speech. Such adverse effects can significantly affect patient weight, mood, and daily functioning [4]. Mucositis is also associated with increased morbidity and mortality in addition to significant additional hospital costs [5], [6].

The pathogenesis of oral mucositis appears to involve five biological phases: (i) initiation, (ii) primary damage response, (iii) signal amplification, (iv) ulceration, and (v) healing [2]. Radiation and chemotherapy lead to the generation of reactive oxygen species (ROS), which in turn activate several signaling pathways in the submucosa and epithelium [7], [8]. In particular, non-DNA injury is initiated by a variety of mechanisms, some of which occur via the generation of ROS and the involvement of superoxide (O_2_^·−^), which is one of the ROS suggested to be involved in the pathogenesis of the onset and progression of oral mucositis [2], [9]. Recent evidence has indicated that ROS such as O_2_^·−^ can also cause oxidative stress by a variety of different mechanisms: lipid peroxidation, apoptosis, DNA damage, protein damage including hyaluronic acid and proteoglycans, and oxidation of pro-inflammatory cytokine release by monocytes and macrophages by depleting intracellular thiol compounds and activating nuclear factor κB [10]–[12]. For the treatment and prevention of oral mucositis concurrent with radiation and chemotherapy for cancer, it is very important to focus on ROS, while considering the above mechanisms. Therefore, many rational approaches including enhanced healing, apoptosis reduction, cytokine reduction, and the prevention of free radical DNA damage have been proposed to manage oral mucositis [13]–[16]. Recently, several drugs such as allopurinol, which inhibits the *in vivo* O_2_^·–^generating system, and O_2_^·−^ dismutase mimetic M40403 have been investigated to treat oral mucositis [17], [18]. However, these strategies are based on the assertion that the generation of O_2_^·−^ is involved in the pathogenesis of oral mucositis. In fact, no direct evidence clarifying the existence of ROS including O_2_^·−^ in oral mucositis has been reported.

Nitroxyl radicals are very useful as spin probes for measuring ROS distribution, oxidative stress, oxygen concentration and redox metabolism by *in vivo* ESR in biological systems [19]. We previously reported on the use of an electron spin resonance (ESR)-based technique for the detection of free radical reactions in biological systems [20]–[23]. It has been reported that nitroxyl radicals, referred to as ‘nitroxyl spin probes’, lose their ESR signal by rapidly reacting with O_2_^·–^ (k = 10^4^–10^5^ M^−1^ s^−1^) and HO^·^ (k>10^9^ M^−1^ s^−1^) [24], [25] in the presence of thiols or NAD(P)H [25], as well as other radicals such as alkyl (k = 10^7^–10^9^ M^−1^ s^−1^) [26] and lipid peroxyl radicals [27]. The signal decay rate of the nitroxyl spin probe provides evidence of ROS generation and changes in the redox status of biological systems [19], [28]. Given this background, the aim of the present study was to demonstrate for the first time directly the presence of ROS and the alteration of redox status involving ROS using *in vivo* ESR spectroscopy with nitroxyl spin probe in 5-FU-induced oral mucositis in hamster cheek pouch for the first time.

## Materials and Methods

### Animals

Four-week-old male Syrian golden hamsters were purchased from Janan SLC, Inc. (Shizuoka, Japan). The animals were housed in groups of three per cage in a room maintained under standardized light (12∶12 h light-dark cycle) and temperature (22±3°C) conditions with free access to food pellets and drinking water. The animals were individually numbered using an ear punch (Finger Loop Ear Punch; British Columbia, Canada) and divided into 4 groups: Group 1, saline control; Group 2, acetic acid control (oral mucositis model with 10% acetic acid alone); Group 3, 5-FU administration; and Group 4, oral mucositis model with 5-FU+acetic acid. The experimental protocol used in this study was approved by the Committee on the Ethics of Animal Experiments of Kanagawa Dental University (Permit Numbers: 258, 259, 503, 505, 506, 508–510) and accorded with the guidelines of the US National Institutes of Health Guide for the Care and Use of Laboratory Animals (NIH Publication No. 85-23, revised 1985). All experiments were performed under sodium pentobarbital anesthesia, and hamsters were euthanized prior to tissue harvesting to minimize suffering.

### Oral Mucositis Induction Protocol

The protocol for the induction of oral mucositis was modified on the basis of a previously published protocol [29], [30]. The 5-FU group and the oral mucositis group were administered an intraperitoneal injection of 5-fluorouracil (5-FU Injection 1000 mg; KYOWA KIRIN, Tokyo, Japan) on days 0 and 2 of the experiment at a dose of 60 mg/kg body weight. On day 2, under sodium pentobarbital (50 mg/kg, *i.p.*) anesthesia, the left side cheek pouch of hamster was extended outside the oral cavity. The central portion of the pouch was held with a 26×1/2″ needle (NEORUS®; TERUMO CORPORATION, Tokyo, Japan) on expanded polystyrene. Subsequently, a micro-syringe with a 29G×1/2″ needle (Myjector®; TERUMO CORPORATION, Tokyo, Japan) was used for the local injection of 10% acetic acid solution (30 µl) under cheek pouch mucosa in the section held by the needle in Groups 2 and 4. The cheek pouch was then returned to the oral cavity, and the animals were returned to their cages. In a similar manner, physiological saline was applied to the lesions in the sham control group.

**Figure 1:**
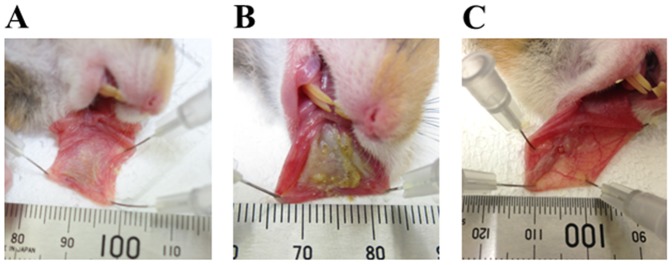
The photograph of oral mucositis in hamster cheek pouch induced by 5-fluorouracil plus acetic acid. (A) Healthy hamster cheek pouch. (B) Oral mucositis appearance on day 4. (C) After treatment with 5-fluorouracil (60 mg/kg, *i.p.*) with 10% acetic acid on day 16.

**Figure 2:**
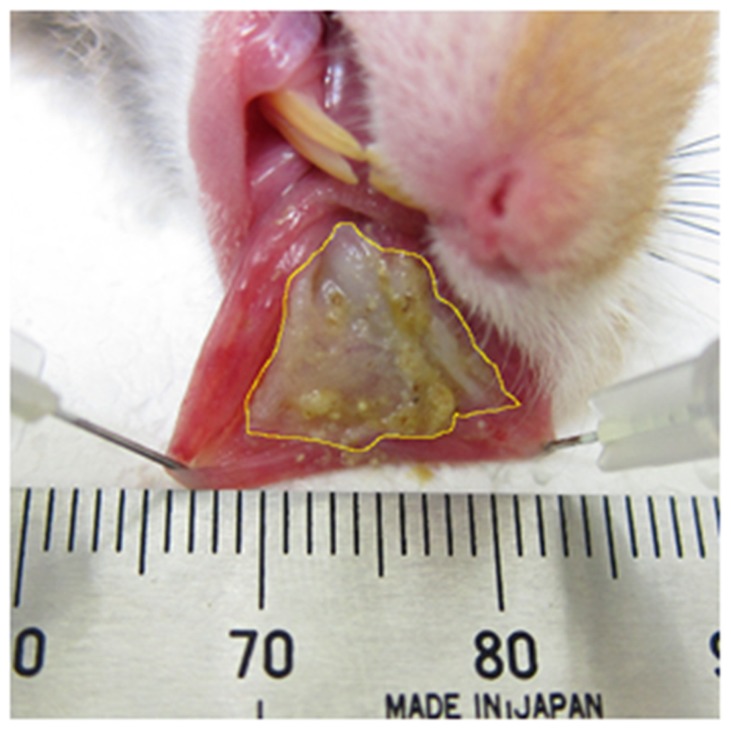
Assessment of the oral mucositis area using a free computer program (Image J, NIH, USA).

All animals were observed and weighed on days 0, 1, 2, 3, 4, 7, 9, 11, 14, and 16. For the purposes of observation (Fig. 1), hamsters were anesthetized with sodium pentobarbital (50 mg/kg, *i.p.*) and the left side cheek pouch was everted and a clinical photograph of the oral mucositis was taken with a digital camera (DSC-RX100; SONY CORPORATION, Tokyo, Japan). In each photograph, a stainless ruler (TZ-1341; KOKUYO Co., Ltd., Osaka, Japan) was included to provide calibration of the mucositis area measurement between images. A free computer program (Image J; NIH, Washington DC, MD, USA) (http://rsbweb.nih.gov/ij/, accessed on 21 May 2009) was used to measure the area of mucositis as cm^2^ (Fig. 2).

### Assay for Lipid Peroxidation of Oral Mucositis in Cheek Pouch

On day 4, hamsters were anesthetized with sodium pentobarbital (50 mg/kg, *i.p.*), the cheek pouch was everted, and the hamsters were killed by cervical dislocation immediately after photographing. After killing the hamsters, the cheek pouch was removed and these specimens were immediately frozen in liquid nitrogen and fractured by applying pulsed pressure with Cryo-Press (Microtec Co., Ltd., Chiba, Japan). Lipid peroxidation was measured as the level of malondialdehyde (MDA) determined by the thiobarbituric acid (TBA) reaction following the manufacturer’s instructions (TBARS Assay Kit; Cayman Chemical Company, Ann Arbor, MI, USA) [31]–[33]. The absorbance was measured at 540 nm. The concentration of MDA is expressed as µM of MDA/mg of protein.

### *In vivo* L-band ESR Analysis

On day 4, we anesthetized the hamsters with pentobarbital as well as performing the TBARS assay. The left side cheek pouch of hamster was extended outside the oral cavity. The central portion of the pouch was held with a 29×1/2″ needle (NEORUS®; TERUMO CORPORATION, Tokyo, Japan) on 3 cm×3 cm expanded polystyrene. The solution of 140 mmol/l carbamoyl-PROXYL (C-PROXYL) was injected into the cephalic vein (10 mg/kg), and placed immediately in an L-band ESR spectrometer (JES-RE-3L; JEOL, Tokyo, Japan) equipped with a 4-window loop-gap resonator. The ESR spectra were repeatedly measured in the left cheek pouch outside the oral cavity of the hamster, beginning 15 sec after injection as described previously [20], [21], [34]. ESR spectra were analyzed using the Win-Rad data analysis system (Radical Research, Tokyo, Japan). The rate constants of the signal decay of nitroxyl were calculated from the signal decay curves, which were determined from semi-logarithmic plots of the peak heights of the ESR signal at a lower magnetic field. All experiments were repeated a minimum of 5 times.

### Statistical Analysis

Analysis of variance and multiple comparison tests using Tukey’s method were applied to determine differences among the 4 groups. Data are expressed as the mean ± SD. Statistical significance was set at *P*<0.05.

## Results

**Figure 3:**
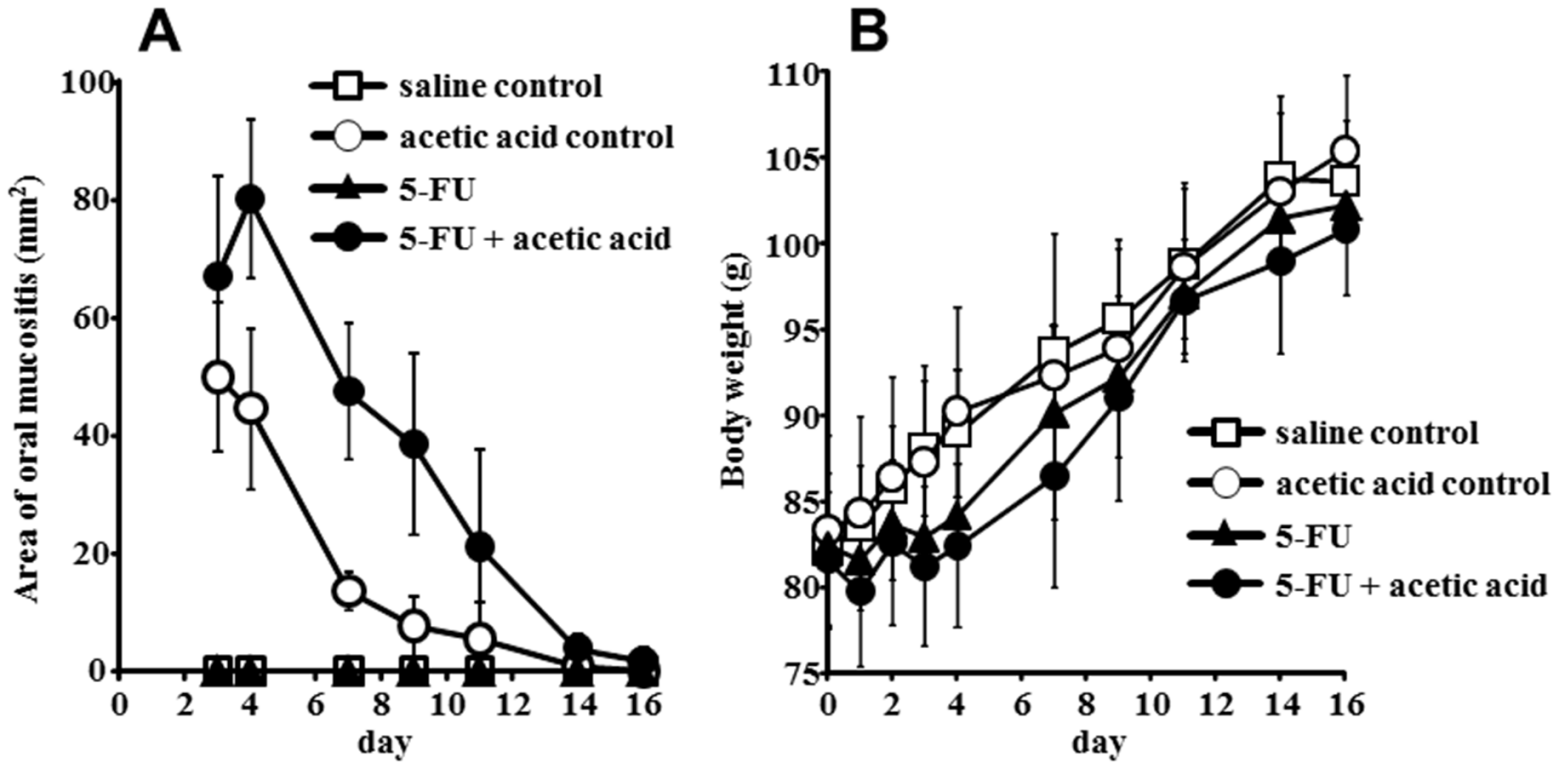
Efficacy of oral mucositis in the hamster model induced by 5-fluorouracil (5-FU) plus acetic acid. A, Changes in oral mucositis area for each treatment group were calculated and plotted over the course of the study. B, Changes in body weight for each treatment group were calculated and plotted over the course of the study. Hamsters received two intraperitoneal injections of 5-FU or saline on days 0 and 2. They were also injected with 10% acetic acid or saline in the left side cheek pouch on day 2. Each point represents the mean ± SD (n = 8–10).

Fig. 1 shows a photograph of oral mucositis induced by 5-FU with acetic acid in hamster cheek pouch. In this model, the combination of intraperitoneal injections of 5-FU and direct treatment of 10% acetic acid on cheek pouch caused oral mucositis on day 3. The area of oral mucositis peaked on day 4 with 5-FU+acetic acid, while it peaked on day 3 in the acetic acid control group (Fig. 3A). Subsequently, the area of oral mucositis became smaller in time-dependent manner and almost complete recovered was observed on day 16. On the other hand, the treatment of 10% acetic acid alone on cheek pouch also induced oral mucositis, like the treatment of the 5-FU+acetic group, but the maximum area was small in comparison with that in the 5-FU+acetic group (Fig. 3A). However, intraperitoneal injection of saline alone and 5-FU treatment alone did not induce oral mucositis. The body weight of hamsters was significantly decreased compared with that in the saline control treatment group on the day after treatment of 5-FU with acetic acid (Fig. 3B).

**Figure 4:**
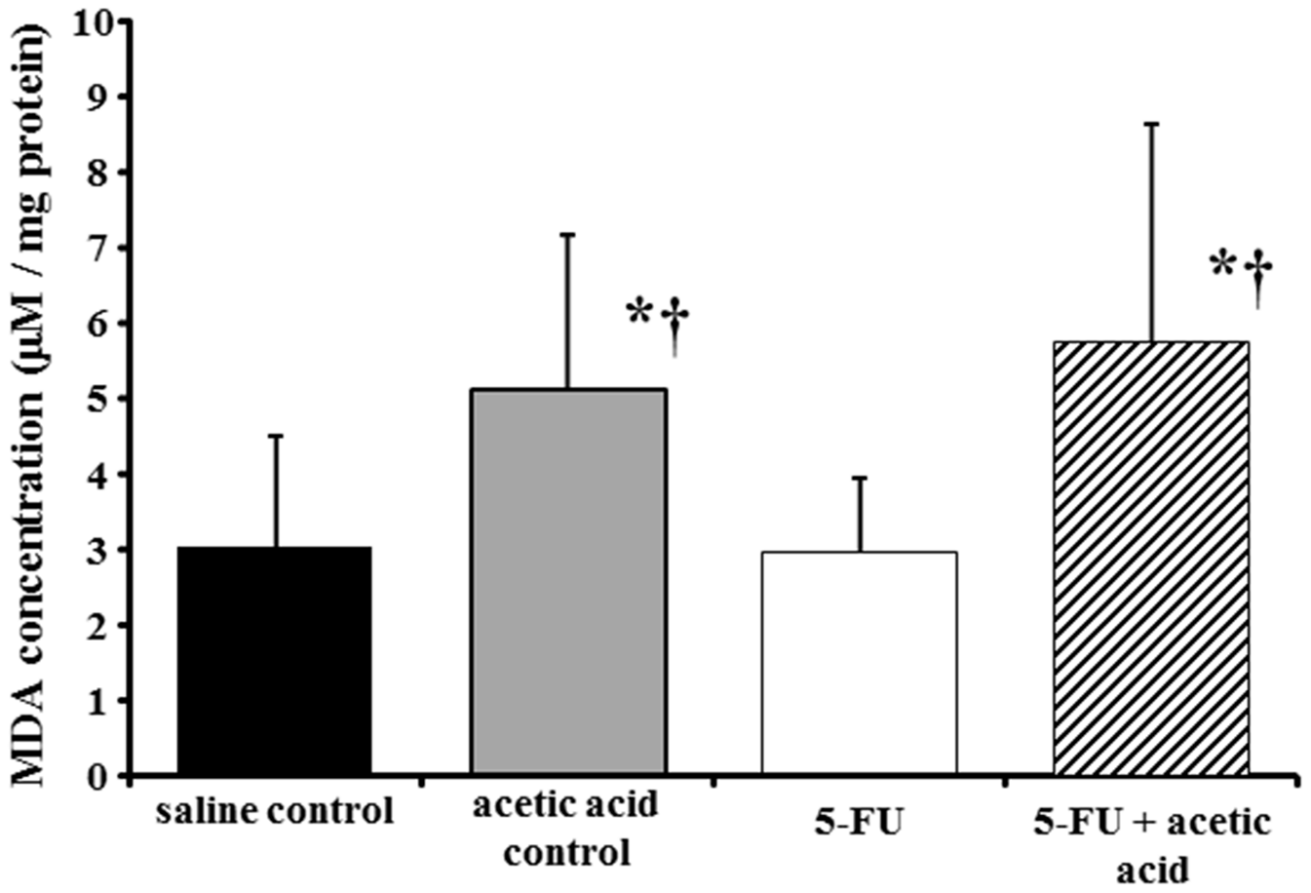
Malondialdehyde (MDA) concentration in the isolated cheek pouch with oral mucositis. MDA concentration was assessed by TBARS assay and normalized by the amount of protein. The data are expressed as mean ± SD in all groups (n = 8–11). Statistical analysis was conducted using Tukey’s method. Experimental conditions are described in *Materials and Methods*. *P<0.05 vs. saline control, †*P*<0.05 vs. 5-FU.

Oxidative stress in the cellular environment results in the formation of highly reactive and unstable lipid hydroperoxides. Decomposition of the unstable peroxides derived from polyunsaturated fatty acids results in the formation of MDA, which can be quantified colorimetrically following its controlled reaction with TBA [31], [33]. We analyzed the level of lipid peroxidation induced in the hamster cheek pouch by the treatment of acetic acid alone and 5-FU with acetic acid. The levels of lipid peroxidation were significantly increased in the oral mucositis groups compared with that in the saline control group (*P*<0.05) (Fig. 4). However, no significant difference was recognized in the 5-FU alone group compared with a saline control group.

**Figure 5:**
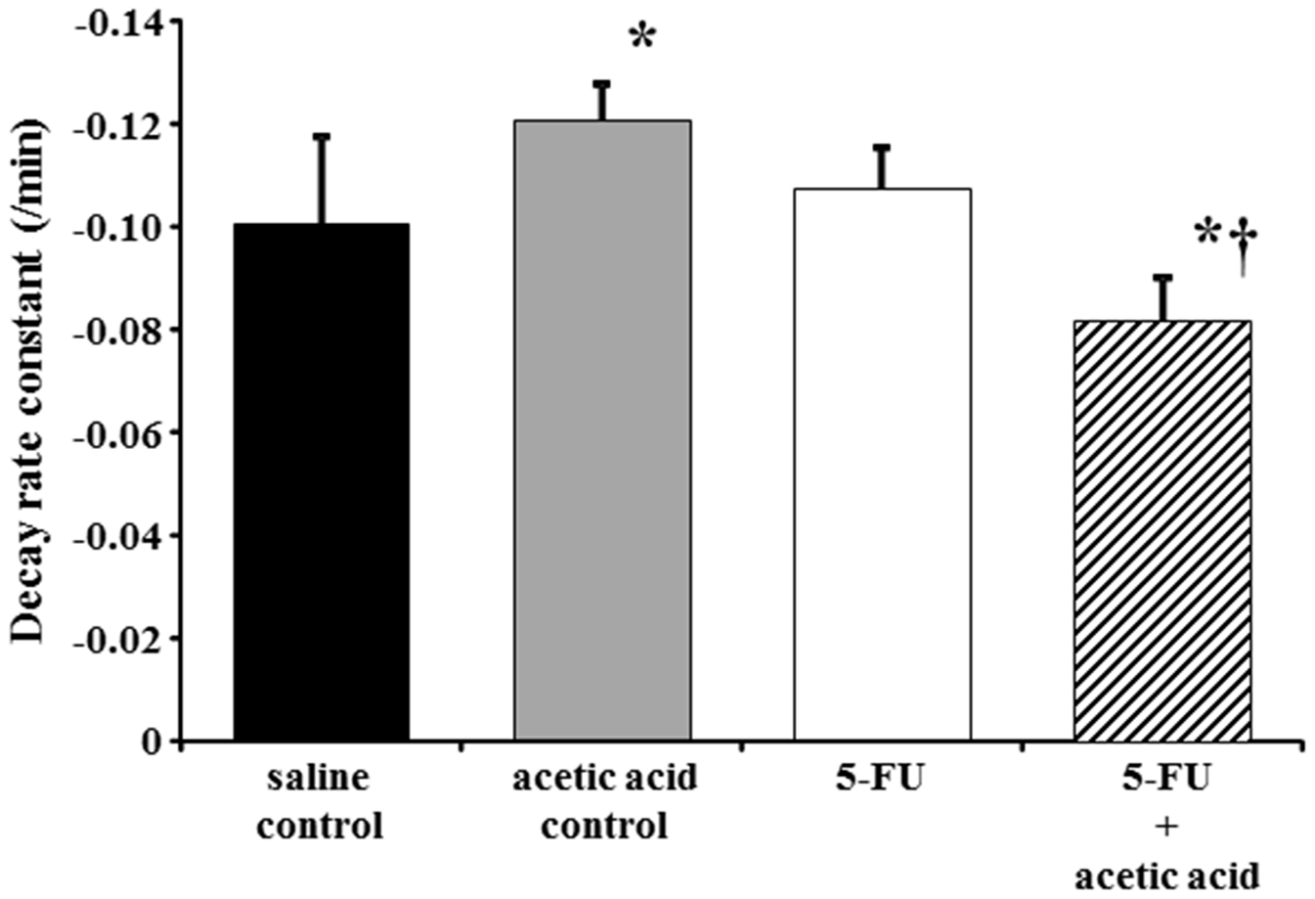
Decay rate constant of carbamoyl-PROXYL (C-PROXYL) in hamster cheek pouch. *In vivo* L-band electron spin resonance spectroscopy was measured at intervals of 15 seconds from 30 seconds after *i.v.* treatment with C-PROXYL. Results are expressed as mean ± SD in all groups (n = 5–8). Statistical analysis was conducted using Tukey’s method. Experimental conditions are described in *Materials and Methods*. *P<0.05 vs. saline control, †*P*<0.05 vs. 5-FU.

C-PROXYL is a suitable spin probe agent for the study of free radical reactions in several tissues by *in vivo* L-band ESR detection [22], [35], [36]. Using this method, we succeeded in measuring oxidative stress as a decay rate constant of C-PROXYL in oral mucositis of hamster cheek pouch (Fig. 5). The signal of C-PROXYL decreased in a time-dependent manner in all experimental groups. In particular, even though the decay rate constant of C-PROXYL in the acetic acid control group was significantly higher than in the saline control group, that in the 5-FU with acetic acid group was lower than those in the other groups (*P*<0.05) (Fig. 5).

## Discussion

The goal of this study was to elucidate directly change of the redox state related to the involvement of ROS in oral mucositis. To date, suitable experimental animal models of oral mucositis have hardly been reported. However, a buccal mucosal ulcer model induced by acetic acid, as described in several reports, has been suggested to be effective for healing studies involving oral mucositis [37], [38]. In addition, we used the hamster cheek pouch, which is suitable for extending tissue outside the oral cavity *in vivo*, a procedure that was necessary to measure ROS in oral mucositis directly in this study (Fig. 1). As a result, the largest area of oral mucositis was induced by treatment with 5-FU+acetic acid in the hamster cheek pouch; it had almost recovered 16 days after the onset of symptoms (Fig. 1 and Fig. 3A). In terms of the weight of hamsters that developed oral mucositis, there were few differences in comparison with the saline control group during the experimental period. Therefore, it was suggested that this 5-FU with acetic acid oral mucositis model might be optimal for direct analysis of the mechanism of oral mucositis as a complication of cancer chemotherapy.

MDA has been widely adopted for a sensitive assay of lipid peroxidation in animal tissues [39]. In this study, we demonstrated the increased peroxidation of lipid membrane and the production of MDA as one of the end products using TBARS assay for analyzing the oxidative stress induced by ROS in association with 5-FU plus acetic acid in cheek pouch (Fig. 4). It is well known that antitumor agents such as mitomycin C and adriamycin are associated with ROS, which have cytotoxic effects on tumor cells [40], [41]. In addition, 5-FU is also recognized to generate ROS in biological systems, which play an important role in cell death mechanisms [42]. In the early stage of inflammation, including in oral mucositis, it has been reported that there is a large amount of ROS such as O_2_^·−^ from neutrophils migrating to the site of inflammation [43]. However, these ROS can be scavenged by antioxidant enzymes such as superoxide dismutase (SOD) and catalase, and antioxidants such as ascorbic acid or the glutathione *in vivo* defense system, resulting in less oxidative damage [44]. Therefore, as a significant increase of MDA levels was observed in oral mucositis groups (acetic acid control and 5-FU+acetic acid), it was suggested that ROS might have been generated above their typical concentration *in vivo* in the locality of the oral mucositis. Furthermore, a significant increase of MDA level by 5-FU treatment alone was not recognized. From this result, rodents including hamsters may be able to maintain their redox balance for ascorbate biosynthesis via a biological mechanism, unlike humans.

We subsequently measured ROS directly using an *in vivo* ESR technique outside the oral cavity of hamster cheek pouch affected by oral mucositis. The decay rate constant of C-PROXYL was significantly increased in the group treated with acetic acid alone. The pharmacokinetics of a nitroxyl compound (C-PROXYL) using *in vivo* L-band ESR was first reported by Berliner and Wan [45]. We also demonstrated that the decay rate constant of C-PROXYL was increased in association with oxidative stress by generating ROS [22], [23], [34]. Therefore, it was suggested that the treatment with acetic acid increased the generation of ROS in a local oral mucositis site. On the other hand, the decay rate constant of C-PROXYL was significant decreased in the group treated with 5-FU with acetic acid, as shown in Fig. 5. The result of this study is the opposite of these previous reports. The animal models that we used in the previously reported studies were chronic oxidation stress models with ROS. The model of oral mucositis caused by 5-FU plus acetic acid in this study involves a particularly severe acute inflammatory response compared with an acetic acid control (Fig. 3A). Excess ROS generation in acute inflammation induces enhancement of the activity of some antioxidants, such as SOD, in local inflammation sites [36], [46]. However, the reducing activity of the organism might be decreased, resulting in consumption of an excess of antioxidants accompanying inflammation. In fact, several studies have reported that the decay rate constant of nitroxyl radicals including C-PROXYL was inhibited; these results were attributed to a decrease in the reducing capacity of the organism with excessive inflammatory responses [28], [47], [48]. The cellular reducing activity against nitroxyl radicals is maintained by intracellular antioxidative molecules including glutathione (GSH) and ascorbates, especially by GSH [49], [50]. GSH is also a key molecule in the bio-reduction of nitroxide spin probes at the cellular or organelle level. Therefore, the findings of this *in vivo* L-band ESR study with treatment of 5-FU+acetic acid indicate the possibility that intracellular antioxidants including GSH had been consumed excessively in the locality of the oral mucositis. However, in terms of oral mucositis in the acetic acid control, an increase of the decay rate constant of C-PROXYL, such as nitroxide compounds, was indicated, and ROS might be unlikely to generate excessive intracellular antioxidant levels, despite an acute inflammatory response. In addition, a decrease in antioxidant activity accompanying the generation of excess ROS might have induced redox imbalance. These findings suggest the possibility of deterioration in the pathology of oral mucositis in that the generation of ROS is further increased by the administration of 5-FU. Hence, these results constitute direct evidence of the presence of ROS at an early stage of oral mucositis, as was previously reported.

## Conclusion

*In vivo* L-band ESR is currently the only method to prove the redox state directly in organisms, tissues, and cells. In this study, we directly indicated the presence of ROS at the beginning of the development of oral mucositis and the alteration of the redox state of inflammation associated with ulcer. These results should support approaches directed at ROS when treating oral mucositis and should be useful for study of the development and effectiveness of novel drug treatments in the future.
